# The Effect of Respiration, pH, and Citrate Co-Metabolism on the Growth, Metabolite Production and Enzymatic Activities of *Leuconostoc mesenteroides* subsp. *cremoris* E30

**DOI:** 10.3390/foods11040535

**Published:** 2022-02-13

**Authors:** Annamaria Ricciardi, Livia Vanessa Storti, Marilisa Giavalisco, Eugenio Parente, Teresa Zotta

**Affiliations:** Scuola di Scienze Agrarie, Alimentari, Forestali ed Ambientali (SAFE), Università degli Studi della Basilicata, 85100 Potenza, Italy; annamaria.ricciardi@unibas.it (A.R.); vanessastorti@libero.it (L.V.S.); marilisa.giavalisco@unibas.it (M.G.); teresa.zotta@unibas.it (T.Z.)

**Keywords:** *Leuconostoc mesenteroides*, aerobiosis, respiration, energy metabolism

## Abstract

*Leuconostoc mesenteroides* includes strains used as starter and/or adjunct cultures for the production of several fermented foods. In this study, the effect of anaerobic and respiratory cultivations, as well as of citrate supplementation and different pH values, was evaluated on growth, biomass, metabolite, and enzymatic activities (pyruvate oxidase, POX; NADH-dependent oxidase, NOX; NADH-dependent peroxidase, NPR) of *Leuconostoc mesenteroides* subsp. *cremoris* E30. We compared the respiration-increased growth rate and biomass production of *Leuc. mesenteroides* E30 to anaerobic cultivation. A supplementation of citrate impaired the growth rate of the respiratory cells. As expected, anaerobic cultures did not consume oxygen, and a similar trend in oxygen uptake was observed in respiratory cultures. The aerobic incubation caused changes in the metabolic pattern, reducing the production of ethanol in favour of acetic acid. Citrate was already exhausted in the exponential phase and did not affect the yields in acetic acid and ethanol. NOX activity increased in the presence of oxygen, while catalase was also detected in the absence of hemin. The absence of H_2_O_2_ suggested its degradation by NPR and catalase. Respiratory cultivation provided benefits (increase in growth rate, biomass, and activity in antioxidant enzymes) for *Leuc. mesenteroides* E30. Therefore, the exploitation of respiratory phenotypes may be useful for the formulation of competitive starter or adjunct cultures.

## 1. Introduction

The genus *Leuconostoc* includes 14 species of great importance in food technology [1]. Some strains of *Leuconostoc citreum*, *Leuc. mesenteroides*, and *Leuc. lactis* have been exploited as starter and/or adjunct cultures to improve the quality and shelf-life of several fermented foods (i.e., kimchi, table olives, sourdough and bakery products, fermented milks, butter, cream, and cheeses) [2,3,4,5]. *Leuconostoc* strains, in fact, may contribute to the texture and aromatic profile of fermented foods by producing exopolysaccharides [6] and diacetyl, acetoin, acetate, ethanol and 2,3-butylenglycol [7]. However, some species (*Leuc. gelidum* subsp. *gasicomitatum*, *Leuc. mesenteroides*, *Leuc. carnosum*) are recognized as spoilage agents of meat, fishery, vegetable products, and ready-to-eat meals, as they impair organoleptic features through off-flavor formation, green discoloration, and the production of slime and CO_2_ [8,9,10,11].

*Leuconostoc* species are oxygen-tolerant anaerobes with heterofermentative metabolisms. The catabolism of carbohydrates through the phosphoketolase pathway, with the formation of acetyl phosphate as a key intermediate, leads to the production of CO_2_, lactic acid, acetic acid, or ethanol, depending on the NADH/NAD^+^ ratio. Heterofermentative species grow poorly when glucose is the sole carbon source, and other sugars, such as sucrose, fructose, and/or maltose, are metabolized for energy production and cell functionality [12]; fructose may be also used as an alternative electron acceptor for the regeneration of reduced NADH. Some members of *Leuc. mesenteroides*, additionally, are able to convert citrate into oxaloacetate and acetate via citrate lyase-oxaloacetate decarboxylase, supplying the intracellular pools of pyruvate and increasing the production of aroma compounds [13,14]. Like other lactic acid bacteria (LAB), some strains of *Leuc. mesenteroides* may tolerate oxygen and grow in aerobic conditions. Plihon et al. [15,16] demonstrated that aerobic cultivation of *Leuc. mesenteroides* improved biomass yield and affected carbon metabolism by shifting ethanol production to acetic acid accumulation.

Several authors [17,18,19] have shown that in some homofermentative LAB, the supplementation with hemin and menaquinone confers further physiological advantages (energy gain, robustness to oxidative and freeze-drying stresses, and synthesis of antioxidant enzymes) compared to unsupplemented aerobiosis.

To date, the respiratory metabolism has been investigated in a few heterofermentative strains of *Levilactobacillus brevis* [20], *Limosilactobacillus reuteri*, and *Levilactobacillus spicheri* [21], confirming the beneficial effects on growth fitness and stress survival. In *Leuc. gasicomitatum* [22] (actually reclassified as *Leuc. gelidum* subsp. *gasicomitatum*) [23], the heme-supplemented aerobiosis had a positive effect on growth rates, biomass accumulation, and the production of aroma compounds. More recently, Zotta et al. [24] verified that potential respiratory phenotypes are distributed in other heterofermentative species and strains, including several members of *Leuc. mesenteroides*. Among them, *Leuc. mesenteroides* E30 showed an improved growth and oxidative stress tolerance, as well as manganese-dependent catalase activity, when cultivated in aerobic and respiratory conditions.

In this study, the effect of the atmosphere of incubation on the fitness of *Leuc. mesenteroides* subsp. *cremoris* E30 has been further investigated. Specifically, the effect of anaerobic and respiratory cultivations, as well as of citrate metabolism and different pH values, has been evaluated on the growth performances (kinetics and biomass yield), metabolites production, and activities of several enzymes involved in the oxygen utilization and oxidative stress tolerance of *Leuc. mesenteroides* subsp. *cremoris* E30 in controlled batch cultivations.

## 2. Materials and Methods

### 2.1. Strains and Culture Conditions

*Leuconostoc mesenteroides* subsp. *mesenteroides* E30 (isolated from raw milk for the production of Canestrato cheese) was maintained as freeze-dried stock in reconstituted 11% (*w*/*v*) skim milk containing 0.1% (*w*/*v*) ascorbic acid, in the culture collection of the Laboratory of Industrial Microbiology, Università degli Studi della Basilicata, and was routinely propagated in WMB pH 6.8 [25] for 16 h at 30 °C.

### 2.2. Fermentation Conditions

The growth of *Leuc. mesenteroides* E30 was evaluated for 24 h at 30 °C in modified WMB (mWMB) with 20 g/L of glucose [26], with or without 5 g/L of sodium citrate, in batch cultivations carried out under anaerobic (AN; nitrogen flow at 0.02 L/min) and respiratory (RS; 15% dissolved oxygen concentration, dO_2_%, supplemented with 1.25 μg/mL of hemin and 1 μg/mL of menaquinone) conditions at pH 5.5 or 6.5, according to a complete 2^3^ factorial design (Table 1).

Bioreactors (3 L working volume for AN and RS conditions, respectively; Applikon, Schiedam, The Netherlands) were inoculated (2% *v*/*v*) with an overnight (16 h, 30 °C) WMB anaerobic pre-culture, washed twice with 20 mM potassium phosphate buffer pH 7 (PB7), and standardized to a final absorbance at 650 nm (A_650_) of 3.0 (Bio-Rad Smart Spec™Plus, Bio-Rad Laboratories Inc., Milan, Italy).

dO_2_% was measured using a polarographic electrode (Applisens, Applikon Biotechnology, Delft, The Netherlands) and was automatically controlled (ezControl controller, Applikon; set point 15%) by varying the stirrer speed (impeller speed from 300 to 800 rpm; two Rushton turbines, 45 mm diameter) and the opening (from 0% to 100%) of the air flow valve (1 *v*/*v*/min maximum air flow). pH was controlled (pH setpoint 5.5 or 6.5) by the automatic addition of a sterile 3:1 NaOH/NaHCO_3_ solution 4 N, while foaming was controlled by the automatic addition of a sterile 10% (*v*/*v*) Antifoam A solution (Fluka, Sigma-Aldrich, St. Louis, MO, USA).

Two independent cultivations were carried out for each growth condition. Samples were aseptically withdrawn at 1 h intervals for the first 9 h and then at 24 h for the measurement of A_650_. At the end of the stationary phase (24 h), a standard curve was generated to correlate A_650_ values to cell dry weight (CDW; washed biomass was dried at 105 °C for 24 h). The parameters of the growth curves were estimated with the dynamic model of Baranyi and Roberts [27] using DMFit v 3.5 for Excel [28].

### 2.3. Oxygen Uptake

Oxygen uptake was measured in standardized (A_650_ = 1) exponential and stationary phase cell suspensions by using a resazurin reduction assay [29] in anaerobiosis and by monitoring (polarographic electrodes; BioXpert 2 software, Applikon) the decrease in oxygen concentration (dO_2_%) every 10 s for 5 min in exponential (A_650_ = 1) and late exponential (9 h) phases in respiration. dO_2_% values were transformed into µmol of O_2_ using Henry’s law [30] and calculating the specific oxygen uptake rate (µmol O_2_/min/g of biomass).

### 2.4. Biochemical Analyses and Enzymatic Activities

Enzymatic kits (R-Biopharm AG, Darmstadt, Germany) were used to quantify the consumption of glucose and citric acid and the production of ethanol and lactic and acetic acids in culture supernatants collected in exponential and stationary phases.

The amounts of H_2_O_2_ in the supernatants, and the activities of pyruvate oxidase (POX), NADH-dependent oxidase (NOX), NADH-dependent peroxidase (NPR), and catalase (CAT) in cell-free extracts (mechanical lysis with FastPrep-24 Instrument, MP Biomedicals, Irvine, CA, USA; 5 cycles of 60 s at speed 6.0) were measured according to Zotta et al. [31], in both exponential and stationary phases. For each biochemical and enzymatic assay, two technical replicates were carried out for each biological condition.

### 2.5. Statistical Analysis

All statistical and graphic analyses were performed using the free software R (https://www.r-project.org/, accessed date on 1 December 2021), version 3.4.2 [32], while package tidyverse [33] was used for tidying data and results and for generating graphs.

## 3. Results

### 3.1. Growth Kinetics and Oxygen Uptake

**Figure 1:**
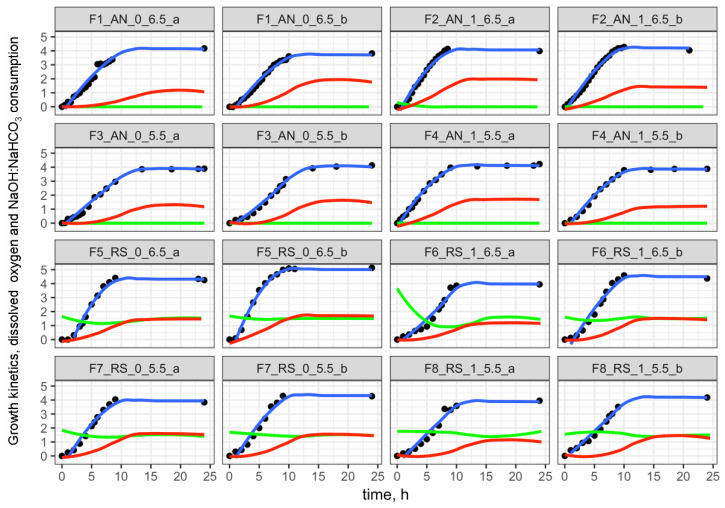
Growth kinetics of *Leuconostoc mesenteroides* E30. The conditions of each fermentation (F1 to F8) are detailed in Table 1. X-axis, time (hours) of incubation; Y-axis, growth (lnX/X0), dissolved oxygen (dO_2_%), and NaOH:NaHCO_3_ (meq/L) consumption; black symbols, biomass production; blue line, kinetics of growth (ln X/X0) estimated with the model of Baranyi and Roberts [27] where X and X0 are, respectively, the biomass measured at each withdrawal time and at start of fermentation; green line, dO_2_% measured through polarographic electrode; red line, milliequivalents/L (meq/L) of NaOH:NaHCO_3_ consumed during fermentation. Code of fermentation name: AN, anaerobiosis; RS, respiration; 0, absence of sodium citrate in mWMB; 1, supplementation of mWMB with 5 g/L of sodium citrate; 5.5, pH controlled at 5.5; 6.5, pH controlled at 6.5.

The kinetics of growth of *Leuc. mesenteroides* E30 was evaluated at different growth conditions (AN vs. RS), carbon source (glucose vs. citrate), and pH values (5.5 vs. 6.5) (Figure 1). The dynamic model of Baranyi and Roberts [27], used to estimate the growth parameters (Table 2), provided a good fit for all cultivations (R^2^ from 0.998 to 0.972).

The maximum specific growth rate (µ_max_) was affected by each examined factor (see Supplementary Table S1 for significance levels). The highest µ_max_ values were found in the respiratory cultures compared to anaerobic ones, regardless of pH setpoint and citrate supplementation. Respiration also affected the duration of the lag phase, which was generally higher than anaerobic cultivations. Citrate *boosted* the growth rate only in anaerobiosis; on the contrary, *its* supplementation decreased the µ_max_ in respiratory conditions. As expected, pH 5.5 impaired the growth rate compared to pH 6.5 (optimal values for cultivation).

**Figure 2:**
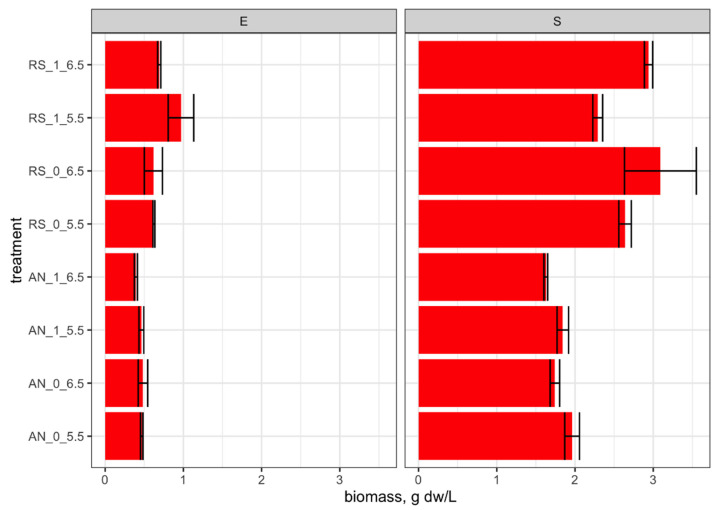
RS, respiratory growth (with 15% dissolved oxygen (dO_2_%), supplementation with 1.25 μg/mL of hemin and 1 μg/mL of menaquinone); AN, anaerobic growth (with nitrogen flow at 0.02 L/min). Conditions of each fermentation (F1 to F8) are detailed in Table 1. Code of fermentation name: AN, anaerobiosis; RS, respiration; 0, absence of sodium citrate in mWMB; 1, supplementation of sodium citrate (5 g/L) in mWMB; 5.5, controlled pH at 5.5; 6.5, pH controlled at 6.5. Growth phase: E, exponential phase; S, stationary phase. Mean values and standard errors are reported.

The production of biomass (Figure 2) was affected by the type of incubation (*p* < 0.001) in both growth phases; interaction between the type of incubation and citrate (*p* < 0.05) influenced the biomass value in the exponential phase, whereas the interaction between the type of incubation and pH (*p* < 0.01) affected the biomass production in the stationary phase (see Supplementary Table S2 for significance levels). Respiratory conditions increased the biomass production compared with anaerobic cultivations, and the most abundant production was measured at pH 6.5 and in the absence of citrate. Cultivation at pH 5.5 had a different effect depending on the type of incubation; in fact, the suboptimal pH promoted the biomass production under anaerobic incubation but impaired it in respiration.

As expected, oxygen consumption was observed only in respiratory cultures; oxygen uptake, however, was not affected by citrate, pH, or growth phase, and no difference in the specific oxygen consumption rate was observed in cells grown under different respiratory conditions.

### 3.2. Consumption of Substrates and Production of Metabolites

Results related to the consumption of substrates and production of metabolites are shown in Table 3. Respiration increased the biomass yield of *Leuc. mesenteroides* E30 compared with anaerobiosis (see Supplementary Table S2 for significance levels). In the exponential growth phase, the strain consumed about 10–40% of glucose initially present in the culture medium, exhausting it completely only in the stationary phase. Citrate was totally consumed already in the exponential phase.

Respiration reduced lactate and ethanol formation and concurrently increased acetate production, compared to anaerobic incubation (see Supplementary Table S3 for significance levels). In all conditions, citrate slightly increased the lactate concentration, whose maximum yield was observed during anaerobic cultivation at pH 6.5. The production of acetate was dependent on the interaction of different factors (i.e., citrate supplementation, pH values, and growth phase). Ethanol, which was an abundant metabolite in anaerobic growing cells, reached the highest concentration when the pH was controlled at pH 6.5 and in the absence of citrate.

### 3.3. Activities of Enzymes Related to Oxygen Metabolism and Oxidative Stress

**Figure 3:**
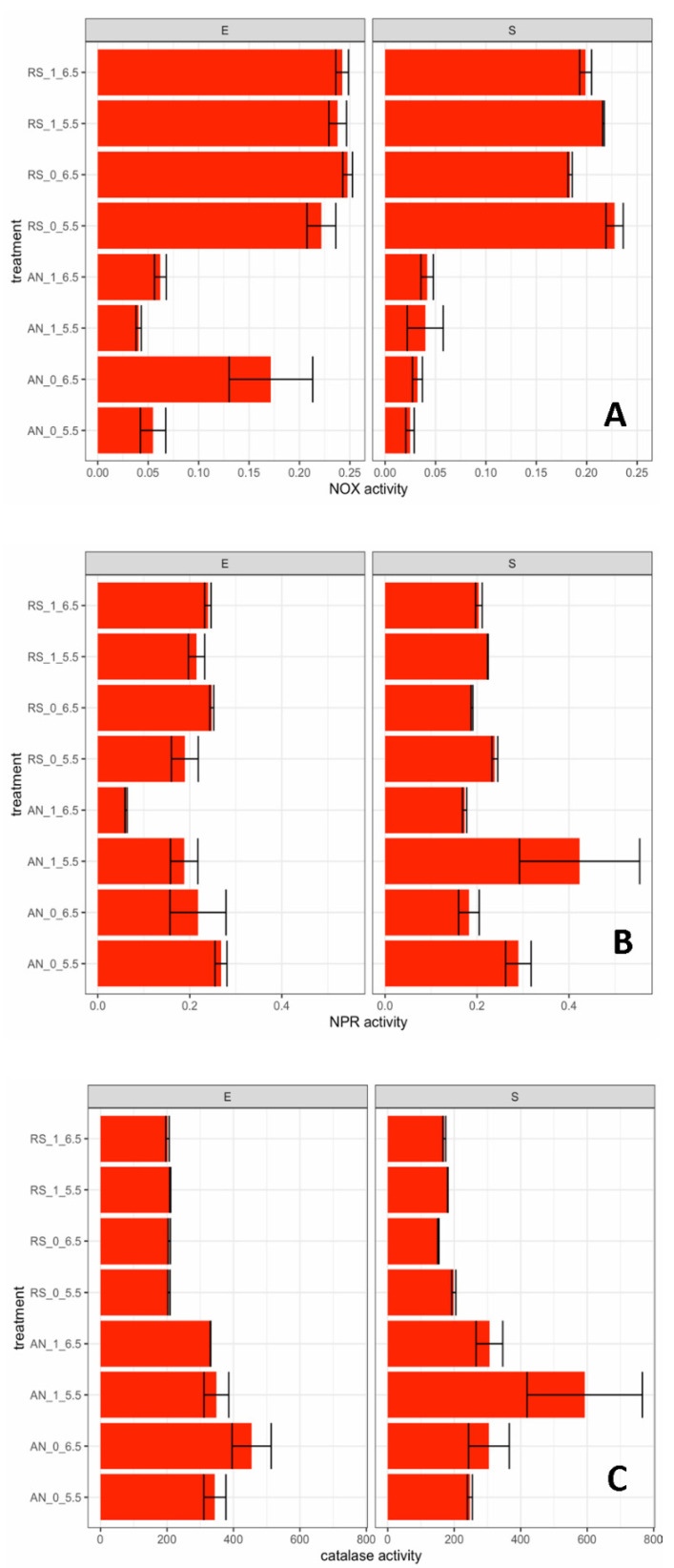
Enzymatic activity of NADH oxidase (NOX, (**A**)), NADH peroxidase (NPR, (**B**)), and catalase (CAT, (**C**)), respectively, expressed in µkatal/mg protein. Growth conditions: RS, respiratory growth (with 15% dissolved oxygen (dO_2_%), supplementation with 1.25 μg/mL of hemin and 1 μg/mL of menaquinone); AN, anaerobic growth (with nitrogen flow at 0.02 L/min). Conditions of each fermentation (F1 to F8) are detailed in Table 1. Code of fermentation name: AN, anaerobiosis; RS, respiration; 0, absence of sodium citrate in mWMB; 1, supplementation of sodium citrate (5 g/L) in mWMB; 5.5, controlled pH at 5.5; 6.5, pH controlled at 6.5. Growth phase: E, exponential phase; S, stationary phase. Mean values and standard errors are reported.

The activity of pyruvate oxidase (POX), the main enzyme of aerobic metabolism, was lower than the sensitivity limit of the assay (0.005 µkatal/mg protein) for all growth conditions. The activities of NADH-dependent oxidase (NOX), NADH-dependent peroxidase (NPR), and catalase (CAT) in exponential (A_650_ = 1) and stationary (24 h of incubation) phases were reported in Figure 3A–C.

Both in exponential and stationary phases, the enzymatic levels of NOX in respiratory cultures were higher (see Supplementary Table S4 for significance levels) than those measured in anaerobic ones. In respiratory conditions, the highest levels of NOX were detected in the exponential growth phase; in the stationary phase, the enzymatic activity was slightly impaired at pH 6.5 regardless of citrate supplementation. In the exponential phase, the activity of NPR was affected (*p* < 0.01) by citrate and by interactions between the type of incubation and citrate and between the type of incubation and pH. In the stationary phase, instead, pH (*p* < 0.01) and interaction between the type of incubation and pH (*p* < 0.05) showed a significant effect on NPR activity (Supplementary Table S4). The catalase activity of *Leuc. mesenteroides* E30 was mainly affected by the type of incubation (*p* < 0.01) in both exponential and stationary growth phases (Supplementary Table S4), and the anaerobically growing cells showed the highest activities. H_2_O_2_ was not detected in any of the tested conditions (data not shown), probably due to its degradation by catalase and NPR activities.

## 4. Discussion

In this study, for the first time, the effect of respiratory cultivation (i.e., oxygen and supplementation with hemin and menaquinone), pH (5.5 and 6.5), and citrate (as an alternative carbon source) was evaluated on the growth, metabolite, and enzymatic activities (POX, NOX, NPR, CAT) of *Leuc. mesenteroides* under controlled batch conditions.

Respiration provided several physiological and metabolic advantages for some LAB (mainly belonging to the homofermentative species *Lactococcus lactis*, *Lactiplantibacillus plantarum*, *Lacticaseibacillus casei* [17,18,19,20]), compared to anaerobic cultivation. In this study, we proved that respiratory cultivation improved some physiological properties also for *Leuc. mesenteroides* E30.

Our data, in fact, demonstrated that the strain was able to perform a respiratory metabolism exhibiting the typical traits of respiratory phenotypes (i.e., increase in growth rates and biomass accumulation and redirection of the pyruvate metabolism towards acetate production). In other heterofermentative LAB, the respiratory growth resulted in physiological benefits (improved biomass, long-term survival, oxygen uptake, changes in carbon metabolism, and the accumulation of acetate and aroma compounds).

Specifically, in *Levilactobacillus spicheri* and *Limosilactobacillus reuteri* cultivated under uncontrolled conditions, the heme-supplemented aerobiosis increased the maximum specific growth rates and the final cell density compared to anaerobic incubation [21]. Similarly, in *Leuc. gelidum* subsp. *gasicomitatum* (former *Leuc. gasicomitatum*), the oxygen and heme, naturally present in meat products, positively affected the growth and biomass yield with important consequences also on the metabolic profile [22]. Oxygen, in fact, represses the expression of alcohol dehydrogenase, thus shifting ethanol to acetate production. However, lactate remains the major metabolite.

Aerobic conversion of pyruvate to acetate could be mediated either by pyruvate dehydrogenase (PDH) or by POX. The latter is the key enzyme for aerobic accumulation of acetate in homofermentative LAB and some heterofermentative LAB, while the effect of PDH on aerobic condition remains unclear [19,21,34]. In *Leuc. mesenteroides* E30, the activity of POX was not found; this could be probably attributed to the presence of the POX gene in the strains of *Leuc. mesenteroides* subsp. *mesenteroides* and subsp. *dextranicum* (IMG database; NCBI database), but not in those belonging to *Leuc. mesenteroides* subsp. *cremoris. Leuc. mesenteroides* E30 was identified at the subspecies level as subsp. *cremoris* ([1], see label M148); therefore, the lack of POX gene is expected.

In our strain, the capability of using oxygen, detoxifying reactive oxygen species (ROS), and coping with oxidative conditions may be due to the presence of other enzymes, such as NOX and NPR. In LAB, NOX and NPR activities contribute to oxidative stress resistance and to intracellular redox balance because these enzymes use NADH to regenerate NAD^+^. Specifically, NOX reacts with oxygen to produce either water or H_2_O_2_. The latter compound is reduced to water by NPR activity. In *Leuc. mesenteroides* E30, NOX and NPR were found in both anaerobic and respiratory cultures; however, the respiratory growth of *Leuc. mesenteroides* E30 remarkably increased NOX activity.

Similarly, an oxygen-induced function of NOX was detected in *Limosilactobacillus panis*. Furthermore, oxygen availability did not affect the gene expression but increased the enzymatic activity with remarkable effects on energy yield and metabolism [35]. In *Fructilactobacillus sanfranciscensis*, the oxygen availability did not affect NOX activity; however, a NOX-negative strain showed a fructose-dependent growth response under aerobic conditions [36].

The fact that NPR activity of *Leuc. mesenteroides* E30 was not affected by the respiratory condition could suggest the presence of only water-forming NOX, as previously described in *Leuc. mesenteroides* subsp. *mesenteroides* by Sakamoto et al. [37]. The absence of H_2_O_2_ in all conditions could prove the effectiveness of the NOX/NPR coupled system in the oxidative stress protection of *Leuc. mesenteroides* E30.

The degradation of H_2_O_2_ in *Leuc. mesenteroides* E30 could be due to catalase activity, even if its production was not induced only by heme supplementation, suggesting possible activity of a manganese-dependent isoform. Heme- and Mn-dependent catalase activities have been previously described in some heterofermentative LAB [21,24,38]. Moreover, some annotated genomes of *Leuc. mesenteroides* exclusively harbor gene encoding for Mn catalase (IMG database).

In this study, the effect of glucose and citrate, as carbon sources, was also evaluated. Specifically, citrate, used in co-metabolism to glucose, affected the biomass production and metabolic production of *Leuc. mesenteroides* E30, depending on the type of incubation. Citrate had a positive impact on the growth performances of anaerobically grown cells, but it impaired those of respiratory cells. In some LAB, citrate may be converted into succinate via the reductive tricarboxylic acid cycle or supply the pool of pyruvate by citrate lyase-oxaloacetate decarboxylase activities [19]. In anaerobic conditions, pyruvate is reduced into lactate by lactate dehydrogenase activity; in aerobic conditions, the accumulation of pyruvate resulting also from citrate metabolism, could interfere with the growth of respiratory cultures. Citrate, instead, had no effect on acetic acid and ethanol yields.

Heterofermentative LAB may use several carbon sources, i.e., maltose, fructose, and sucrose [12], including citrate, with a direct effect on their physiology [36]. Özcan et al. [39], through an in silico study, observed a stimulation of *Leuc. mesenteroides* growth during the co-utilization of citrate and glucose, in agreement with other experimental data [40,41]. The presence of citrate, contributing to the pyruvate pool through oxaloacetate decarboxylase, caused an increased production of lactate and, consequently, an increased oxidation of NADH. Therefore, because the requirement of ethanol production for the re-oxidation of NADH decreases, citrate contributes to the acetate pool by acetate kinase activity with additional ATP yield.

To date, the potential of respiratory phenotypes has been industrially exploited only by the CHR Hansen Company (Denmark) to improve the production of a lactococcal starter culture (direct-to-vat F-DVS pHageControl™ R-604). The benefits of the respiratory metabolism, –, are different. Furthermore, in previous studies, Reale et al. [42,43] demonstrated that some strains of *Lacticaseibacillus casei* cultivated in respiratory conditions affected the biochemical and organoleptic features of some foods (i.e., reduction in oxidative processes, increase in secondary proteolysis and changes in the aroma compound in Cheddar-type cheeses and in sourdoughs).

*Leuc. mesenteroides* plays a positive role in the production of several fermented foods. The capability to produce gas, exopolysaccharides, and several aroma compounds, in fact, may contribute to the development of a desired texture and flavour in different products (e.g., fermented vegetables, bakery products, and some cheese varieties). Therefore, the understanding of metabolic pathways and the exploitation of strains with improved phenotypic features and stress resistance, also for this species, may be of practical relevance for different food-related applications.

## 5. Conclusions

This study demonstrated that respiratory cultivation improved the growth rate and biomass production, and affected the metabolic pattern, energetic state, and activity of antioxidant enzymes of *Leuc. mesenteroides* E30, compared to anaerobic conditions.

Therefore, the respiratory phenotype of *Leuc. mesenteroides* E30 could be exploited as a starter and/or an adjunct culture to improve the organoleptic and nutritional properties of several foods, especially vegetable- and cereal-based products.

## Figures and Tables

**Table 1:** Experimental design used to evaluate the effect of the type of incubation, carbon source, and pH on growth, biomass, metabolites production, and enzymatic activities.

Fermentation	Incubation ^a^	Citrate ^b^	pH
F1	AN	0	6.5
F2	AN	1	6.5
F3	AN	0	5.5
F4	AN	1	5.5
F5	RS	0	6.5
F6	RS	1	6.5
F7	RS	0	5.5
F8	RS	1	5.5

**^a^** AN, anaerobic growth with nitrogen flow at 0.02 L/min; RS, respiratory growth with 15% dissolved oxygen (dO_2_%), supplementation with 1.25 μg/mL of hemin and 1 μg/mL of menaquinone. **^b^** 0, absence of sodium citrate in mWMB; 1, supplementation of mWMB with 5 g/L of sodium citrate.

**Table 2:** Parameters of growth kinetics of *Leuconostoc mesenteroides* E30.

Growth Condition	µ_max_ (h^−1^)	lag (h)	R^2^ of Fit
F1_AN_0_6.5_a	0.46 ± 0.02	0.00 ± 0.00	0.972
F1_AN_0_6.5_b	0.46 ± 0.01	1.38 ± 0.21	0.996
F2_AN_1_6.5_a	0.64 ± 0.01	1.05 ± 0.11	0.998
F2_AN_1_6.5_b	0.57 ± 0.01	1.13 ± 0.14	0.997
F3_AN_0_5.5_a	0.40 ± 0.02	1.92 ± 0.25	0.995
F3_AN_0_5.5_b	0.42 ± 0.02	3.09 ± 0.22	0.997
F4_AN_1_5.5_a	0.50 ± 0.01	0.00 ± 0.00	0.997
F4_AN_1_5.5_b	0.47 ± 0.01	1.54 ± 0.19	0.997
F5_RS_0_6.5_a	0.77 ± 0.03	2.17 ± 0.19	0.997
F5_RS_0_6.5_b	0.82 ± 0.06	0.36 ± 3.57	0.992
F6_RS_1_6.5_a	0.72 ± 0.07	4.58 ± 0.32	0.988
F6_RS_1_6.5_b	0.71 ± 0.03	2.58 ± 0.21	0.995
F7_RS_0_5.5_a	0.63 ± 0.03	2.11 ± 0.18	0.996
F7_RS_0_5.5_b	0.67 ± 0.03	2.41 ± 0.20	0.996
F8_RS_1_5.5_a	0.55 ± 0.06	3.24 ± 0.50	0.974
F8_RS_1_5.5_b	0.49 ± 0.03	3.00 ± 0.35	0.988

Growth condition: RS, respiratory growth (with 15% dissolved oxygen (dO_2_%), supplementation with 1.25 μg/mL of hemin and 1 μg/mL of menaquinone); AN, anaerobic growth (with nitrogen flow at 0.02 L/min). Conditions of each fermentation (F1 to F8) are detailed in Table 1. Code of fermentation name: AN, anaerobiosis; RS, respiration; 0, absence of sodium citrate in mWMB; 1, supplementation of mWMB with 5 g/L of sodium citrate; 5.5, controlled pH at 5.5; 6.5, pH controlled at 6.5; µ_max_, maximum specific growth rate; lag (h), duration of lag phase; R^2^ of fit quantified the goodness of modeling.

**Table 3:** The consumption of glucose and citrate and the production of metabolites of *Leuconostoc mesenteroides* E30.

Growth Condition ^a^	Growth Phase ^b^	Biomass Yield ^c^	Glucose Consumed ^d^	Lactic Acid Yield ^e^	Acetic ACID Yield ^f^	Ethanol Yield ^g^
F1_AN_0_6.5	E	0.074 ± 0.032	34.8 ± 3.356	0.595 ± 0.248	0.038 ± 0.010	0.450 ± 0.0186
	S	0.072 ± 0.009	131.93 ± 4.512	0.979 ± 0.043	0.019 ± 0.021	0.682 ± 0.046
F2_AN_1_6.5	E	0.102 ± 0.070	27.16 ± 21.253	1.707 ± 1.339	1.205 ± 0.978	0.158 ± 0.065
	S	0.076 ± 0.006	116.81 ± 5.761	1.209 ± 0.070	0.196 ± 0.012	0.666 ± 0.108
F3_AN_0_5.5	E	0.074 ± 0.005	32.43 ± 0.746	0.763 ± 0.003	0.036 ± 0.024	0.606 ± 0.074
	S	0.082 ± 0.009	130.78 ± 1.361	0.843 ± 0.001	0.010 ± 0.003	0.653 ± 0.087
F4_AN_1_5.5	E	0.140 ± 0.006	17.01 ± 2.424	1.706 ± 0.278	1.530 ± 0.176	0.247 ± 0.185
	S	0.082 ± 0.006	122.02 ± 2.834	1.186 ± 0.287	0.177 ± 0.038	0.590 ± 0.148
F5_RS_0_6.5	E	0.232 ± 0.036	13.71 ± 4.847	0.802 ± 0.186	0.726 ± 0.181	0.090 ± 0.098
	S	0.135 ± 0.051	126.43 ± 1.510	0.613 ± 0.011	0.634 ± 0.008	0.130 ± 0.146
F6_RS_1_6.5	E	0.145 ± 0.022	24.78 ± 1.491	0.924 ± 0.164	0.000 ± 0.000	0.000 ± 0.000
	S	0.128 ± 0.004	124.88 ± 1.920	0.783 ± 0.069	0.611 ± 0.028	0.105 ± 0.059
F7_RS_0_5.5	E	0.077 ± 0.027	43.77 ± 14.169	0.244 ± 0.088	0.147 ± 0.073	0.000 ± 0.000
	S	0.113 ± 0.005	126.91 ± 4.754	0.468 ± 0.070	0.499 ± 0.022	0.089 ± 0.097
F8_RS_1_5.5	E	0.455 ± 0.174	11.21 ± 0.559	1.877 ± 0.053	1.649 ± 0.094	0.000 ± 0.000
	S	0.102 ± 0.033	122.41 ± 4.661	0.940 ± 0.009	0.469 ± 0.025	0.100 ± 0.008

**^a^** Growth conditions: RS, respiratory growth (with 15% dissolved oxygen (dO_2_%), supplementation with 1.25 μg/mL of hemin and 1 μg/mL of menaquinone); AN, anaerobic growth (with nitrogen flow at 0.02 L/min). The conditions of each fermentation (F1 to F8) are detailed in Table 1. Code of fermentation name: AN, anaerobiosis; RS, respiration; 0, absence of sodium citrate in mWMB; 1, supplementation of mWMB with 5 g/L of sodium citrate; 5.5, controlled pH at 5.5; 6.5, pH controlled at 6.5. **^b^** Growth phase: E, exponential phase; S, stationary phase. Mean values of two biological replicates ± standards errors are reported. **^c^** Biomass yield: calculated as (X-X_0_)/(S_0_-S), i.e., biomass production (X-X_0_), g/L, relative to consumed glucose (S_0_-S), g/L. **^d^** Glucose consumed: consumed glucose (S_0_-S), mM. **^e^** Lactate yield: production of DL-lactic acid (P-P_0_), g/L, relative to consumed glucose (S_0_-S), g/L. **^f^** Acetate yield: production of acetic acid (A-A_0_), g/L, relative to consumed glucose (S_0_-S), g/L. **^g^** Ethanol yield: production of acetic acid (E-E_0_), g/L, relative to consumed glucose (S_0_-S), g/L.

## Data Availability

Not applicable.

## Supplementary Materials

The following are available online at https://www.mdpi.com/article/10.3390/foods11040535/s1, Table S1. Analysis of variance on the effect of atmosphere of incubation, pH, citrate and their interactions on specific growth rate (μmax), Table S2: Analysis of variance on the effect of atmosphere of incubation, pH, citrate and their interactions; Table S3. Analysis of variance on the effect of atmosphere of incubation, pH, citrate and their interactions; Table S4. Analysis of variance on the effect of atmosphere of incubation, pH, citrate and their interactions.
