# Supplementary material for: The Effect of Respiration, pH, and Citrate Co-Metabolism on the Growth, Metabolite Production and Enzymatic Activities of Leuconostoc mesenteroides subsp. cremoris E30
Source: Foods. 2022 Feb 13;11(4):535. doi: 10.3390/foods11040535 (PMC8871477; doi:10.3390/foods11040535)
Supplement: Supplementary file 1 [file foods-11-00535-s001.zip › foods-1545020-supplementary.pdf]

Title

Effect of respiration, pH and citrate co-metabolism on the growth metabolite production and enzymatic activities of *Leuconostoc mesenteroides* subsp. *cremoris* E30

Authors

Annamaria Ricciardi[1], Livia Vanessa Storti[1,#], Marilisa Giavalisco[1], Eugenio Parente [1,*] and Teresa Zotta[1]

Affiliations

[1] Scuola di Scienze Agrarie, Alimentari, Forestali ed Ambientali (SAFE), Università degli Studi della Basilicata, 85100 Potenza, Italy; annamaria.ricciardi@unibas.it; vanessastorti@libero.it; marilisa.giavalisco@unibas.it; eugenio.parente@unibas.it; teresa.zotta@unibas.it

* Corresponding author

Prof. Eugenio Parente

E-mail: eugenio.parente@unibas.it Tel.: +39-0971-205561

[#] Affiliation to the time of work

Supplementary Table S1. Analysis of variance on the effect of atmosphere of incubation, pH, citrate and their interactions on specific growth rate (μ_{max}).

	Sum sq	Mean sq	F value	Pr (>F)
**ATM**	0.128	0.128	121.766	4.05e-06***
**CIT**	0.000	0.000	0.026	0.875
**pH**	0.065	0.065	61.894	4.92e-05***
**ATM:CIT**	0.044	0.044	42.177	1.89e-04***
**ATM:pH**	0.007	0.007	6.658	0.032*
**CIT:pH**	0.004	0.004	3.599	0.094
**ATM:CIT:pH**	0.000	0.000	0.194	0.671

*** indicated $p<0.001$; ** indicated $p<0.01$; * indicated $p<0.05$.

ATM, atmosphere of incubation (respiration vs anaerobiosis); CIT, citrate (presence vs absence); pH, (6.5 vs 5.5); ATM:CIT, interaction between atmosphere of incubation and citrate; ATM:pH, interaction between atmosphere of incubation and pH; CIT:pH, interaction between citrate and pH; ATM:CIT:pH, interaction between atmosphere of incubation, citrate and pH.

Supplementary Table S2. Analysis of variance on the effect of atmosphere of incubation, pH, citrate and their interactions on biomass production and yield, in exponential and stationary phases.

		Exponential phase				Stationary phase			
		**Sum sq**	**Mean sq**	**F value**	**Pr (>F)**	**Sum sq**	**Mean sq**	**F value**	**Pr (>F)**
Biomass production	**ATM**	0.598	0.598	25.935	3.29e-05***	7.165	7.165	59.100	6.34e-08***
	**CIT**	0.053	0.053	2.287	0.143	0.271	0.271	2.235	0.148
	**pH**	0.057	0.057	2.501	0.127	0.222	0.222	1.830	0.189
	**ATM:CIT**	0.132	0.132	5.747	0.025*	0.036	0.036	0.293	0.593
	**ATM:pH**	0.027	0.027	1.166	0.291	1.184	1.184	9.764	0.005**
	**CIT:pH**	0.064	0.064	2.770	0.109	0.020	0.020	0.163	0.690
	**ATM:CIT:pH**	0.017	0.017	0.743	0.397	0.019	0.019	0.156	0.696
Biomass yield	**ATM**	0.067	0.067	13.875	0.006**	0.007	0.007	18.864	0.002**
	**CIT**	0.037	0.037	7.629	0.025*	0.000	0.000	0.117	0.741
	**pH**	0.009	0.009	1.949	0.200	0.000	0.000	0.717	0.422
	**ATM:CIT**	0.010	0.010	1.982	0.197	0.000	0.000	0.334	0.579
	**ATM:pH**	0.003	0.003	0.709	0.424	0.001	0.001	2.805	0.132
	**CIT:pH**	0.063	0.063	13.011	0.007**	0.000	0.000	0.047	0.834
	**ATM:CIT:pH**	0.046	0.046	9.372	0.015*	0.000	0.000	0.002	0.967

*** indicated $p<0.001$; ** indicated $p<0.01$; * indicated $p<0.05$.

ATM, atmosphere of incubation (respiration vs anaerobiosis); CIT, citrate (presence vs absence); pH, (6.5 vs 5.5); ATM:CIT, interaction between atmosphere of incubation and citrate; ATM:pH, interaction between atmosphere of incubation and pH; CIT:pH, interaction between citrate and pH; ATM:CIT:pH, interaction between atmosphere of incubation, citrate and pH.

Supplementary Table S3. Analysis of variance on the effect of atmosphere of incubation, pH, citrate and their interactions on the yields of lactic acid, acetic acid an ethanol, in exponential and stationary phases.

		Exponential phase				Stationary phase			
		**Sum sq**	**Mean sq**	**F value**	**Pr (>F)**	**Sum sq**	**Mean sq**	**F value**	**Pr (>F)**
Lactic acid yield	**ATM**	0.213	0.213	0.850	0.383	0.499	0.499	40.188	2.23e-4***
	**CIT**	3.632	3.632	14.491	0.005**	0.370	0.370	29.827	6.01e-4***
	**pH**	0.079	0.079	0.314	0.590	0.005	0.005	0.434	0.529
	**ATM:CIT**	0.023	0.023	0.090	0.772	0.001	0.001	0.096	0.765
	**ATM:pH**	0.013	0.013	0.051	0.827	0.007	0.007	0.591	0.464
	**CIT:pH**	0.451	0.451	1.799	0.217	0.043	0.043	3.464	0.100
	**ATM:CIT:pH**	0.706	0.706	2.815	0.132	0.009	0.009	0.714	0.423
Acetic acid yield	**ATM**	0.021	0.021	0.159	0.700	0.821	0.821	1652.206	1.48e-10***
	**CIT**	2.954	2.954	22.815	0.001**	0.021	0.021	42.798	1.80e-4***
	**pH**	0.484	0.484	3.742	0.089	0.023	0.023	46.637	1.34e-4***
	**ATM:CIT**	0.889	0.889	6.864	0.031*	0.040	0.040	79.758	1.96e-05***
	**ATM:pH**	0.140	0.140	1.079	0.330	0.015	0.015	31.017	5.3e-4***
	**CIT:pH**	1.632	1.632	12.607	0.007**	0.000	0.000	0.145	0.713
	**ATM:CIT:pH**	0.904	0.904	6.982	0.030*	0.000	0.000	0.004	0.950
Ethanol yield	**ATM**	0.636	0.636	38.214	2.65e-4***	1.173	1.173	121.458	4.09e-06***
	**CIT**	0.205	0.205	12.337	0.008**	0.002	0.002	0.224	0.649
	**pH**	0.002	0.002	0.128	0.730	0.006	0.006	0.589	0.465
	**ATM:CIT**	0.039	0.039	2.350	0.164	0.001	0.001	0.115	0.744
	**ATM:pH**	0.039	0.039	2.342	0.164	0.001	0.001	0.087	0.776
	**CIT:pH**	0.000	0.000	0.007	0.936	0.000	0.000	0.003	0.961
	**ATM:CIT:pH**	0.006	0.006	0.361	0.564	0.002	0.002	0.178	0.684

*** indicated p<0.001; ** indicated p<0.01; * indicated p<0.05.

ATM, atmosphere of incubation (respiration vs anaerobiosis); CIT, citrate (presence vs absence); pH, (6.5 vs 5.5); ATM:CIT, interaction between atmosphere of incubation and citrate; ATM:pH, interaction between atmosphere of incubation and pH; CIT:pH, interaction between citrate and pH; ATM:CIT:pH, interaction between atmosphere of incubation, citrate and pH.

Supplementary Table S4. Analysis of variance on the effect of atmosphere of incubation, pH, citrate and their interactions on the activities of NADH-dependent oxidase (NOX), NADH-dependent peroxidase (NPR) and catalase (CAT), in exponential and stationary phases.

		**Exponential phase**				**Stationary phase**			
		**Sum sq**	**Mean sq**	**F value**	**Pr (>F)**	**Sum sq**	**Mean sq**	**F value**	**Pr (>F)**
NOX	**ATM**	0.193	0.193	170.835	2.09e-12***	0.237	0.237	929.719	<2e-16***
	**CIT**	0.006	0.006	5.690	0.025*	0.000	0.000	1.664	0.209
	**pH**	0.014	0.014	12.643	0.002**	0.001	0.001	5.441	0.028*
	**ATM:CIT**	0.009	0.009	8.042	0.009**	0.000	0.000	0.807	0.378
	**ATM:pH**	0.006	0.006	5.179	0.032*	0.002	0.002	9.929	0.004**
	**CIT:pH**	0.007	0.007	6.052	0.021*	0.000	0.000	0.853	0.365
	**ATM:CIT:pH**	0.003	0.003	2.408	0.134	0.000	0.000	2.013	0.169
NPR	**ATM**	0.012	0.012	4.073	0.055	0.023	0.023	2.460	0.130
	**CIT**	0.024	0.024	8.030	0.009**	0.008	0.008	0.829	0.372
	**pH**	0.004	0.004	1.445	0.241	0.091	0.091	9.877	0.004**
	**ATM:CIT**	0.032	0.032	10.733	0.003**	0.008	0.008	0.831	0.371
	**ATM:pH**	0.033	0.033	11.221	0.003**	0.041	0.041	4.494	0.044*
	**CIT:pH**	0.006	0.006	2.038	0.166	0.006	0.006	0.690	0.414
	**ATM:CIT:pH**	0.001	0.001	0.296	0.591	0.015	0.015	1.615	0.216
CAT	**ATM**	214159	214159	71.347	1.19e-08***	279880	279880	15.730	0.001***
	**CIT**	7218	7218	2.405	0.134	60109	60109	3.378	0.078
	**pH**	3643	3643	1.214	0.281	40970	40970	2.303	0.142
	**ATM:CIT**	6940	6940	2.312	0.141	60107	60107	3.378	0.078
	**ATM:pH**	5012	5012	1.670	0.209	14864	14864	0.835	0.370
	**CIT:pH**	9393	9393	3.129	0.090	47577	47577	2.674	0.115
	**ATM:CIT:pH**	7081	7081	2.359	0.137	72134	72134	4.045	0.055

*** indicated p<0.001; ** indicated p<0.01; * indicated p<0.05.

ATM, atmosphere of incubation (respiration vs anaerobiosis); CIT, citrate (presence vs absence); pH, (6.5 vs 5.5); ATM:CIT, interaction between atmosphere of incubation and citrate; ATM:pH, interaction between atmosphere of incubation and pH; CIT:pH, interaction between citrate and pH; ATM:CIT:pH, interaction between atmosphere of incubation, citrate and pH.
